# Effect of Bamboo biochar on strength and water retention properties of low plastic clay and silty sand

**DOI:** 10.1038/s41598-023-33466-8

**Published:** 2023-04-17

**Authors:** Shailesh Kumar Yadav, Ramakrishna Bag

**Affiliations:** Department of Civil and Environmental Engineering, Indian Institute of Technology Patna, Patna, Bihar India

**Keywords:** Core processes, Geochemistry, Geomorphology, Environmental impact

## Abstract

Biochar is a carbon-rich stable product derived from the thermochemical decomposition of biomass. The properties of biochar vary with types of feedstock, heating rate, pyrolysis temperature, etc. Consequently, the mechanical and hydrological properties of biochar amended soil (BAS) also differ with types of biochar and soils. However, the effect of bamboo biochar (BB) amendment on soil strength and water retention properties is missing in the previous literature. Bamboo biomass was pyrolysed at 600 °C to produce biochar. BB and soils (low plastic clay (CL) and silty sand (SM)) were mixed to prepare BAS. The samples were prepared by mixing BB in five ratios, i.e., 0%, 1%, 2%, 3.5% and 5% of dry soil weight. The biochar application has increased optimum moisture content, alkalinity (pH) and Atterberg limits, whereas, reduced maximum dry density and specific gravity of both the soils (CL and SM). The unconfined compressive strength (UCS) of CL soil was noted to increase by 10.5% with 2% biochar content and decreased after that, whereas the UCS of SM soil was found to decrease continuously with the biochar content increment. Therefore, the unconfined compressive strength (UCS) result showed that biochar application has contrary effects on both soils. The measured gravimetric water content (GWC) of BAS was increased with biochar increment in both soils. However, GWC increased more in CL than in SM soil at the same biochar content. The microstructural analysis showed that the biochar amendment filled the pore space of the soil matrix, resulting in an increase in UCS and GWC values. The increased water retention capacity and strength (UCS) of biochar amended CL soil provides evidence that it could be used as a landfill cover material.

## Introduction

Biochar (BC) is a carbon-rich stable product derived from the pyrolysis or gasification of biomass through high-temperature with little to no available oxygen in a closed chamber^1,2^. The primary raw materials (feedstock) for biochar production are agricultural residue, animal manure, municipal solid waste, forestry, and wood processing waste^3^. In general, pyrolysis is the thermochemical decomposition of organic matter in an anaerobic environment at a temperature range of 200–700 °C, producing syngas, bio-oil, and biochar^4^. Generally, syngas and bio-oil are used in energy generation^2,5^. Whereas, because of the advantageous properties of biochar, which include higher specific surface area, cation exchange capacity, carbon content, pH, water retention capacity and lower density etc. It is extensively used in the removal of heavy metal contamination, carbon sequestration, agriculture and engineering fields^6,7^. Therefore, biochar has been potentially applied in agriculture, green roofs, bioengineered slopes, embankments, landfills etc. Recently, researchers have investigated the potential applications of biochar as a feasible cover material in landfills and vegetated slope stability^8–16^.

In the past, various studies have been carried out on different types of feedstock-produced biochar to investigate the geotechnical (mechanical and hydrological) properties of biochar amended soil (BAS), and most studies have concluded that biochar properties vary with feedstock types, pyrolysis temperature, rate of heating, and atmospheric condition of pyrolysis chamber^8,17–23^. Moreover, few studies have reported that the BAS properties change with the age of biochar^24^. The mechanical and hydrological properties of BAS also vary with the size of biochar particles^25–27^.

In general, a decrease in the dry density of BAS was reported by researchers^12,27^. Whereas unconfined compressive strength (UCS) and shear strength of BAS contradicted the results, which showed variation with feedstock and soil type. Studies on clayey soil show that UCS value increased up to a certain percentage of biochar content and, after that, it decreased^28,29^. Bora et al.^22^ reported an unaltered or decreased UCS value of silty sand due to increased biochar content. Ahmed et al.^30^ observed a decrease in shear strength on silty loam soil attributed to the biochar addition. Sadasivam and Reddy^15^ and Xu et al.^31^ reported an increase in shear strength parameters owing to biochar application on silty clay and purely clay soil. In addition to mechanical characteristics, biochar application also affects water holding capacity of soil. It has been reported that biochar application enhanced the water retention capacity (WRC) of the soil^21,32–36^. However, some researchers have reported contradictory results, such as biochar application is ineffective in increasing or different feedstock having unalike WRC of BAS^37–40^.

Unlike other cash crops, grass and timber species; bamboos are fast-growing indigenous material with faster maturation and higher productivity. Bamboos require initial plantation costs with no additional expenditure during it’s growth period^41,42^. In addition to this, bamboos have been widely used in developing countries like India, Malaysia and China; as a support and bearing member in fencing, roofing, construction and crafts^43^. This biomass is extensively present in large quantities worldwide, where India is the second-largest producer, with 11.4 million-hectare of the entire forest cover. However, the current effective utilization of bamboo is around 30–40%, and the remaining bamboo turns out to be waste, which is incinerated or buried directly^43^.

Kumar et al.^42^ and Nath et al.^43^ reported that around 4.5 million tons of bamboo are used in industries for various purposes, generating a tremendous aggregation of bamboo scrap or waste materials at the end of the work. The continuous increase in the large quantity of bamboo waste causes a scarcity of dumping space, leading to a severe environmental and waste management problem. To address these challenges, recycling the waste bamboo into biochar is essential and environment-friendly^1^. The production of biochar would be beneficial as well as economical in dealing with such waste.

It is evident from the literature that the mechanical and hydrological properties of BAS vary with feedstock and soil types. Therefore, the investigation of bamboo biochar effect on the mechanical and hydrological properties of BAS is required to understand its efficacy. Hence an attempt has been made to assess the impact of BB modification on the critical mechanical and hydrological properties, such as unconfined compressive strength, compaction characteristics and water retention capacity of soils. The current study also focuses on the potential of bamboo biochar as an amendment material for engineering projects. This research would contribute to the selection of an effective and optimum quantity of biochar for soil amendment. Moreover, the current research would significantly contribute to evaluating the mechanical and hydrological properties of BAS and help further study BB as an amendment material for other types of soils.

## Materials and methodology

### Soil

**Figure 1:**
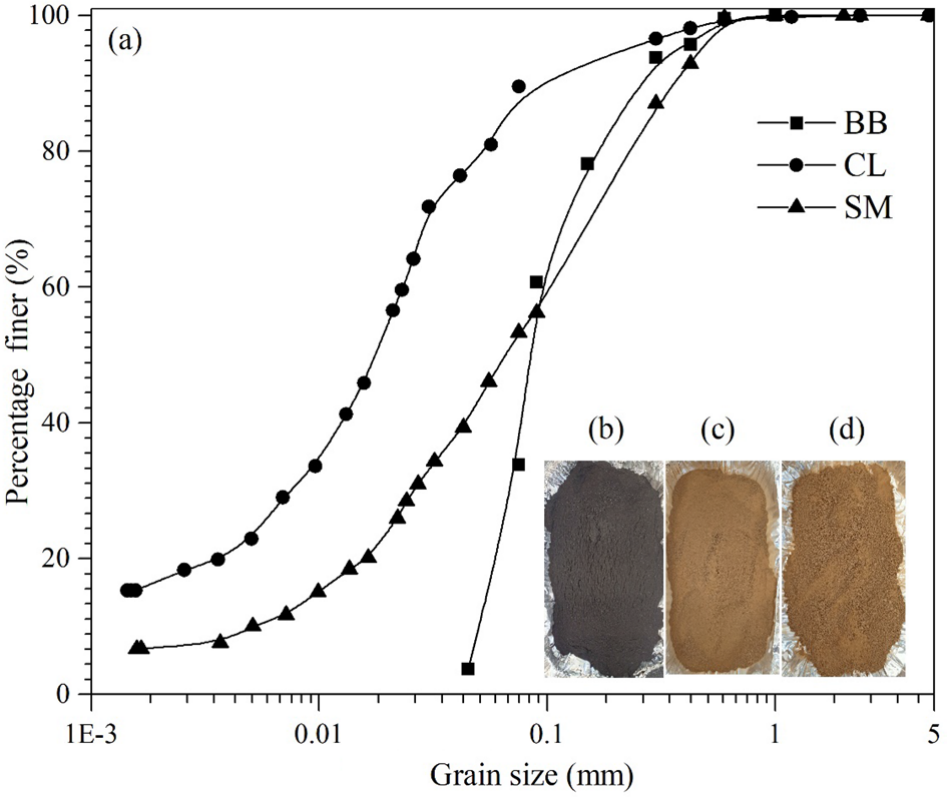
(**a**) Grain size distribution curve of bamboo biochar (BB) and soil samples, (**b**) Photograph of bamboo biochar, (**c**) CL and (**d**) SM soil.

In the current study, two different types of soils were used. The soil samples were collected from two places in Patna district of Bihar, India and are shown in Fig. 1c,d, respectively. In order to collect the sample, topsoil containing grass was removed. After that, a pit was excavated approximately 1 m below the ground surface, and soil samples were filled in bags. Soil samples were brought to the laboratory and spread on a mat, allowed to air dry. Furthermore, the impurities, such as grassroots, stones and pebbles, were separated from soil samples. The samples were crushed with a hammer and ground to make powder and passed through a 2.36 mm sieve. Then the powder soil samples were stored in a closed container for tests.

### Biochar

The bamboo biochar (Fig. 1b) used in this study was procured from VR International Organic Farming Solution, Bhopal, India. The organisation has used bamboo biomass as feedstock material. The procured biochar was pyrolysed at 650–700 °C temperatures. The biochar was stored in an airtight closed container after receiving it from the supplier. Biochar was used as procured from the supplier and was not crushed. After mixing soil and the required quantity of biochar, it was kept in a sealed container for 7 days for homogenization.

## Materials characterization

Characterization of soils was carried out to determine the basic and index properties. Particle size distribution, Atterberg limits, specific gravity, pH of soil and biochar were determined according to the procedure outlined in ASTM standards^44–48^.

Furthermore, Field Emission Scanning Electron Microscopy (FESEM) studies were carried out to examine the surface morphology of soils and biochar. FESEM images were captured at different magnifications ranging from 500X to 25KX using Sigma-300, Zeiss, Germany. For better interpretation, biochar was pre-coated with gold to provide electrical conductivity. In addition, Elemental analyses were also carried out with the Energy Dispersive X-ray spectroscopy (EDS) technique, equipped with FESEM. The mineralogical analysis was performed by X-ray diffraction (XRD) technique using MiniFlex 300/600, United Kingdom. All the soils and biochar samples were scanned for 2θ angle between 5 and 80°, with Cu Kα (λ = 0.154 nm), at a scan rate of 2° per minute and a step size of 0.2°.

Fourier transform infrared spectroscopy (FTIR) was used to investigate the functional groups present in soil and biochar with respect to potassium bromide (KBr) pellet as a reference using FTIR DR-8000 spectrophotometer (Shimadzu Co., Japan). The scan was performed in the range of 4000–400 cm^−1^. The total specific surface area (SSA) of soils and biochar was determined by the ethylene glycol monomethyl ether (EGME) adsorption and desorption method^49^.

### Preparation of soil-biochar composite sample 

The soil samples were placed in an oven for 24 h at 105 °C to remove the soil moisture. Biochar procured from the industry was also dried. After that, dry soils and biochar were hand-mixed thoroughly for 15 min in an aluminium pan and kept in a desiccator. The biochar percentages were decided based on literature-reported results^20,50,51^. Soil samples were mixed with 1, 2, 3.5 and 5% of biochar to prepare BAS. A series of standard Proctor tests on both soils with varying percentages of biochar content were carried out to determine its effect on compaction characteristics^52^, unconfined compressive strength^53^ and water retention^54^ properties of the soil. One series of tests were carried out on the soils (CL and SM) without adding biochar in order to compare the result.

### Determination of compaction characteristics

Dry unit weight of biochar, untreated soils and BAS samples were determined by the Proctor compaction test. All compaction tests were performed by the procedure mentioned in ASTM D698^52^. Before the test, samples were mixed with a definite amount of water content for each case, and the wet samples were kept in a desiccator for 24 h to achieve homogeneity. After that, compaction tests were performed in the laboratory. The bulk weight of the specimen was measured after completing the compaction test, and then the moisture content of the specimen was determined. Representative samples of the compacted specimens were taken at three designated locations (top, center and bottom) and kept in an oven for 24 h at 105 °C temperature to determine the moisture contents. Then, bulk unit weight (ratio of bulk weight of specimen and volume of mould) and moisture content were used to calculate the dry unit weight of compacted specimens. The dry unit weight of specimen was calculated by using the following formulas:

$$ {\text{Dry}}\,{\text{unit}}\,{\text{weight}} = {\text{Bulk}}\,{\text{unit}}\,{\text{weight}}/\left( {{1} + {\text{moisture}}\,{\text{content}}} \right) $$

$$ {\text{Dry}}\,{\text{density}} = {\text{Dry}}\,{\text{unit}}\,{\text{weight}}/{9}.{81} $$

At last, the moisture content and dry density were plotted on the x and y-axis, respectively. From the graph, the maximum dry density (MDD) and optimum moisture content (OMC) were determined. The moisture content corresponding to MDD was considered OMC for the respective samples (ASTM D698^52^). Similarly, three independent samples were tested for each case to minimize error and validate the results.

### Specimen preparation for unconfined compressive strength test 

The unconfined compressive strength (UCS) tests of non-amended and BAS soils were performed according to the procedure mentioned in ASTM D2166^55^. The soils without biochar and soil-biochar mixture were taken from the container and mixed thoroughly to prepare the sample for the UCS test. After that, a standard mould of size 38 mm in diameter and 76 mm in height was selected to prepare the specimen for UCS. The weight of the UCS specimen was determined by volume and unit weight relationship for each sample. The weighted samples were mixed with OMC and placed in a desiccator for 24 h by packing them in a plastic bag to achieve moisture equilibrium. Thereafter, the moist sample was statically compressed in a cylindrical mould from both ends with a manually operated UCS sampler. The UCS specimen was allowed to achieve equilibrium for 5 min after that, it was extracted to perform the UCS test. Similarly, other UCS specimens were also prepared following the same method.

In order to determine the UCS, the prepared specimen was placed vertically on a loading frame of capacity 50 kN procured from Aimil, India. Then, the load plunger and displacement indicator were carefully placed on the specimen. After that, an incremental load was applied to produce axial deformation at a rate of 2.5 mm/minute, and the displacement of the specimen corresponding to the applied load was recorded. The loading was continued until the failure of the specimen took place. Three tests were conducted on identical specimens for the reliability of the results. An average value of three tests was reported as the UCS of the sample.

### Specimen preparation for soil-water retention test

The water retention capacity (WRC) test was performed using WP4C dew-point chilled mirror potentiometer device (Decagon Devices Inc., USA). WP4C dew point chilled mirror potentiometer accurately measures suction in a higher suction range of − 0.1 to − 300 MPa, however, at the lower suction range below − 0.1 MPa, there may be some inaccuracy. This device calculates suction indirectly using Kelvin equation by balancing the relative humidity of the specimen and air present above the specimen in the sealed chamber^54^.

The suction test of bare soil and BAS were carried out in unconfined (slurry) conditions. A round cup of 4 cm diameter and 1 cm depth was selected. Non-treated soil and BAS samples in the slurry form (containing water content equal to liquid limits) were placed in the round cup (mould) without overburden pressure. The slurry surrounding the mould was wiped out, and the excess slurry above the cup was trimmed and cleaned carefully.

The wet sample mould was placed in a sealed box containing silica gel to dry, and once the sample reached the unsaturated stage or the lower suction range, it was taken out for the suction test. After that, the specimen was placed in the WP4C device for suction measurement. The instrument displayed total suction and temperature readings on the liquid crystal display (LCD). Then the sample cup was removed from the equipment and weighed simultaneously. After that, the mould was kept at a controlled temperature to dry. After a few minutes (approximately 10–20 min), the mould was again placed back in the WP4C device to measure suction. This process continued till suction reached 0–40 MPa. After that, silica gel was used to further dry the specimen for higher suction (40–280 MPa). The suction value was noted to increase as the drying of the sample continued in silica gel. This process was continued until the suction value became constant. When the difference between consecutive suction values was noted to be very small, the test was terminated, and the sample’s water content was measured by oven drying.

## Results and discussion

### Properties of soil and biochar

The particle size distribution (PSD) of soil and biochar is shown in Fig. 1a. The analysis of the PSD curve of soil1 indicates that most of the soil particles are silty (73%) and categorized as lean clay (CL) by the unified soil classification system (USCS). Whereas soil2 contains sand (52%), followed by silt (39%), and was classified as silty sand (SM). These soils have been largely used as landfill cover materials in countries like India, USA and China^56,57^. The analysis of the BB particle size distribution curve (Fig. 1a) illustrates coarser particles than CL soil. In contrast, SM soil has both smaller and larger particles than biochar.

**Figure 2:**
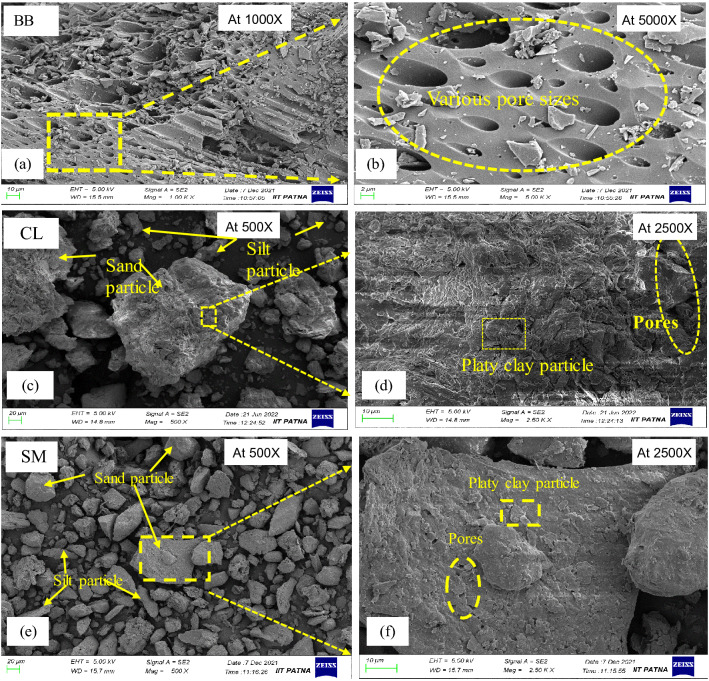
Surface morphology of (**a**, **b**) bamboo biochar; (**c**, **d**) CL and (**e**, **f**) SM soil at different magnifications in powder condition.

The results of basic and index properties of soils and biochar measured in the laboratory as per ASTM standards are summarised in Table 1. The Atterberg limits and specific gravity of CL soil were found to be more than SM soil. Platy and angular shape particles facilitated these properties in the CL than SM soil system (Fig. 2c,d,e,f). Moreover, BB showed a higher liquid limit (108.3%) and lesser specific gravity (1.61) than both soils. It is attributed to the honeycomb structure of biochar (Fig. 2a,b), which holds more water in the available rod-shaped pores. The rod-shaped pores on the surface of BB (sizes ranging > 2 µm) attract nutrients and enhance the WRC in the soil matrix^58–60^. The microstructural analysis also depicts that platy clay particles were stuck over the angular and sub-angular silt particles. The pH of bamboo biochar (8.9) was observed to be more alkaline than the soils (7.75–7.85). The increased pH in biochar is due to the higher carbon content and surface functional groups (hydroxide, alkali and carbonate) (Fig. 4a) of BB^61^. Therefore, BB has the potential to treat acidic soil. The compaction result observed a higher MDD in the case of SM soil than the CL soil (Table 1), and the MDD of BB was observed to be lesser compared to both soils. The lightweight and porous structure caused lesser MDD in BB.

**Table 1:** Basic physical properties, classification, fineness and compaction characteristics of soils and biochar used in the study.

Property	Soil1	Soil2	BB	Standards
Liquid limit (%)	38.15	24.9	108.30	ASTM D4318
Plastic limit (%)	19.5	15.3	….	ASTM D4318
Plasticity index (%)	18.64	9.6	….	ASTM D4318
Classification	CL	SM	….	ASTM D2487
Specific gravity	2.78	2.75	1.61	ASTM D854
pH value	7.75	7.85	8.90	ASTM D4972
OMC (%)	17.25	11.50	70	ASTM D698
MDD ( g/cc)	1.805	2.028	0.676	ASTM D698
SSA (m^2^/g)	52.72	21.21	209.16	(Cerato & Lutenegger, 2002)

*BB* bamboo biochar, *OMC* optimum moisture content, *MDD* maximum dry density, *SSA* specific surface area, *CL* low plastic clay, *SM* silty sand.

**Figure 3:**
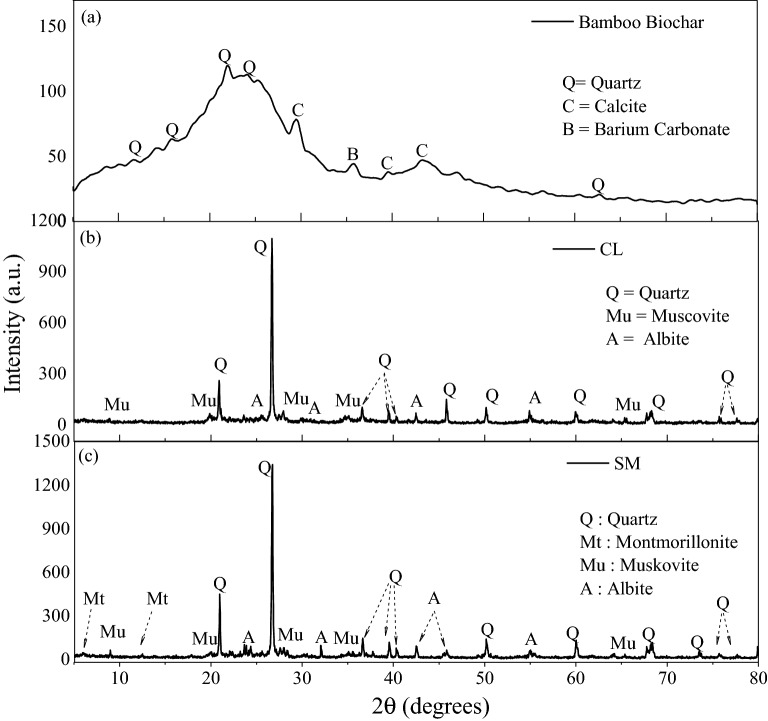
X-ray diffraction analysis of pure (**a**) bamboo biochar, (**b**) CL and (c) SM soil in powder form.

Figure 3a shows the XRD results of bamboo biochar, which confirmed the presence of quartz (SiO_2_), calcite (CaCO_3_), and barium carbonate (BaCO_3_) minerals present in the sample^23,62^. The XRD results of CL and SM soil are shown in Fig. 3b,c, respectively. The spectral analysis of CL soil showed the presence of Quartz (SiO_2_), muscovite (KAl_3_Si_3_O_10_(OH)_2_) and aluminium phosphate (AlPO_4_) minerals^63^. Similarly, the XRD result of SM soil showed the presence of albite (NaAlSi_3_O_8_), montmorillonite (CaAl_4_Si_8_O_24_), muscovite (KAl_3_Si_3_O_10_(OH)_2_) and Quartz (SiO_2_) minerals^27^. Moreover, quartz and muscovite were the predominant minerals in the soils. The peaks corresponding to 5.99° and 12° indicate the presence of montmorillonite minerals in SM soil (Fig. 3c). In general, the montmorillonite mineral improves water absorption and plasticity of the soil. The diffraction peak at 8.98° suggests the presence of muscovite minerals. It is the most common form of mica and helps absorb water into the soil. Albite minerals showed their presence at 23.73°, 32.05°, 42.54°, and 45.71° over the XRD spectra. It belongs to the plagioclase feldspar group and is a good source of plant nutrients. Quartz is a predominant and stable mineral that reduces the water-holding capacity and increases the infiltration of water in the soil.

**Figure 4:**
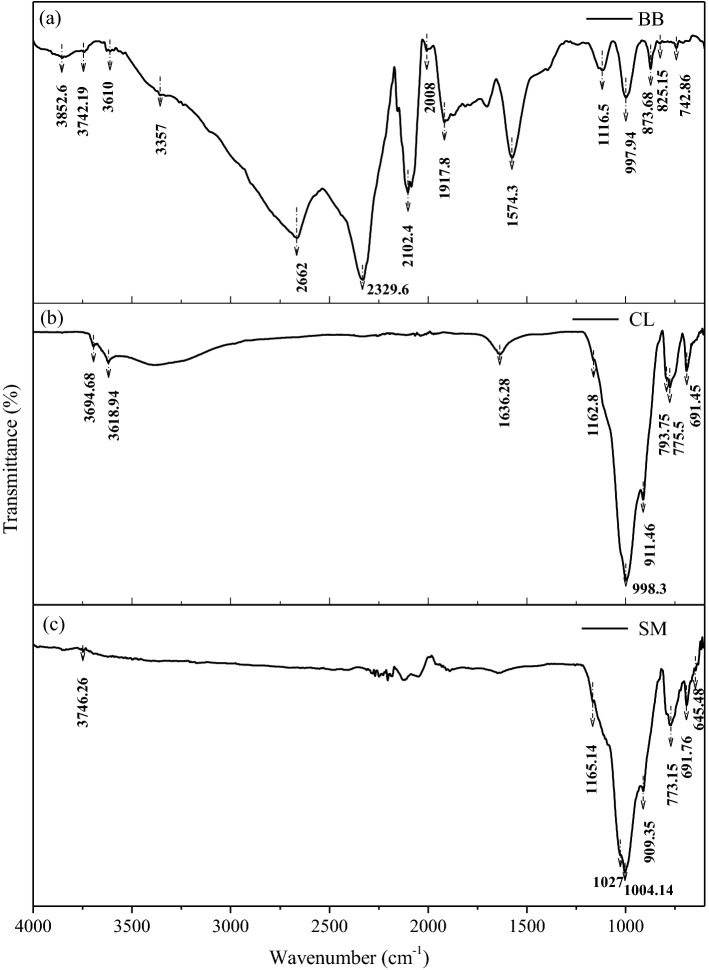
FTIR spectra analysis for powder sample of pure (**a**) bamboo biochar, (**b**) CL and (**c**) SM soil in powder form.

Figure 4a shows the FTIR spectra of bamboo biochar. The spectrum analysis depicts the various active surface functional groups in the band range of 4000–400 cm^−1^. Spectral absorption peaks in bands 700–900, 997, 1116, 1242 and 1392 cm^−1^ show aromatic C–H vibration, C–H stretching, C–OH bending, C–C stretching, and C–O bending, respectively^64^. The higher wavenumber range 4000–2500 cm^−1^ shows the O–H group, commonly present in soil and biochar. The band range 2000–1500 cm^−1^ indicates the presence of the double bond functional group (C=C and C=O), while peaks in 2500–2000 cm^−1^ show the presence of triple bonds^65–67^.

Moreover, the FTIR spectral analysis of CL and SM soil is shown in Fig. 4b,c. Quarz is abundant in the soil in the form of silicate minerals. The regions 400–800 and 900–1100 cm^−1^ shows bending and stretching of the Si–O bond over the spectra, confirming the presence of silicate minerals. The absorption peaks corresponding to the wavenumbers at 3694, 1890, 1162–65, 1004, 998, 909–11, 773–75, 691 and 645 cm^−1^ confirm the presence of a single bond group (Al–O, Si–O, Al–O–Al) in both soil. The peaks corresponding to 1648 and 1636 cm^−1^ indicates water molecules^68^.

### Effect of biochar on physicochemical properties of soils

Table 2 summarises the results of specific gravity, liquid limits, plastic limits and pH of biochar amended soil (BAS). Specific gravity (G_S_) of BAS was found to be decreased from 2.78 (0% BC) to 2.69 (5% BC) for CL; and from 2.75 (0% BC) to 2.65 (5% BC) for SM soil. The continuous reduction in Gs of BAS occurred because lighter and porous biochar particles replaced the heavy soil particles having higher specific gravity. The investigation of Reddy et al.^9^ and Huang et al.^23^ showed similar findings with biochar amendment.

**Table 2:** The variation of specific gravity and physiochemical properties of soil with the increase in biochar content.

Soil-biochar mixture											
Dry weight (%)		CL + BC					SM + BC				
Soil	BC	G_S_	LL(%)	PL(%)	PI(%)	pH	G_S_	LL(%)	PL(%)	PI(%)	pH
100	0	2.78	38.15	19.51	18.64	7.75	2.75	28.50	19.72	8.78	7.85
99	1	2.76	39.20	21.43	17.77	7.82	2.73	29.62	20.83	8.79	7.88
98	2	2.74	40.02	21.96	18.06	7.95	2.70	30.24	21.88	8.36	7.96
96.5	3.5	2.71	40.21	22.30	17.91	8.02	2.68	31.63	23.64	7.99	8.04
95	5	2.69	40.52	23.17	17.35	8.15	2.65	32.47	25.34	7.13	8.17

BC = biochar, G_S_ = specific gravity, LL = liquid limit, PL = plastic limit, PI = Plasticity index.

In adverse to specific gravity, liquid limits of BAS increased with biochar addition in both soils. The liquid limits were observed 38.15% at 0% BC and 40.52% at 5%BC for CL soil, whereas 28.50% at 0% BC and 32.47% at 5%BC for SM soil. The increased liquid limit in BAS occurred due to biochar’s high intra-pores and hydrophilic nature, which enhances the water storage in the mixture.

Likewise, plastic limits also increased from 19.51% (0% BC) to 23.17% (5% BC) for CL and 19.72% (0% BC) to 25.34% (5% BC) for SM soil with biochar addition. The plasticity result shows that biochar increment in soils has modified the fineness of the mixture and increased specific surface area, causing water adsorption, thus enhancing the plastic property (plasticity) of soils. The pH of BAS is an important factor that impacts mineral precipitation, methanotrophic activity, and greenhouse gas emission in landfill cover systems^69,70^. The pH value of BAS increased from 7.75 (0% BC) to 8.15 (5% BC) for CL; and 7.85 (0% BC) to 8.17 (5% BC) for SM soil (Table 2).

### Effect of biochar on OMC and MDD of soil

**Figure 5:**
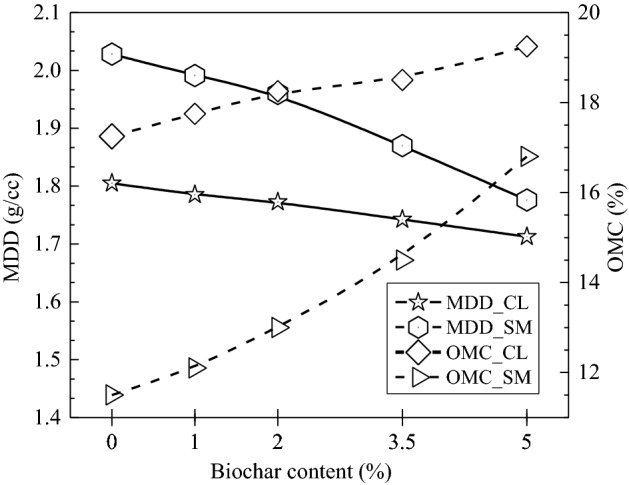
Variation of MDD and OMC of CL and SM soil with the increase in biochar content from 0 to 5% (w/w).

Figure 5 shows the variation of MDD and OMC with the biochar content of both soils. The observation showed that MDD decreased with biochar addition in both soils. The observed decrease in MDD corresponds to 0, 1, 2, 3.5 and 5% biochar content were1.80, 1.78, 1.77, 1.74 and 1.71 g/cc for CL soil; and 2.02, 1.98, 1.95, 1.86 and 1.77 g/cc for SM soil, respectively. Whereas the increase in OMC for 0, 1, 2, 3.5 and 5% biochar content were 17.25, 17.75, 18.25, 18.5 and 19.25% for CL; and 11.5, 12.1, 13, 14.5 and 16.8% for SM soil, respectively. Therefore, it was observed that biochar increment decreased MDD and increased OMC of both soils.

However, the reduction in MDD was more in CL than in SM soil. It occurred because biochar addition created void space and decreased the weight of BAS in the system (Fig. 2a). It also enhanced water and air entrapment in the matrix (due to the surface functional group observed on FTIR spectra), facilitating a slippery surface at the interface of soil biochar composite leading to a decrease in MDD. A similar pattern was observed in Kumar et al.^12^ investigations of BAS with peanut, sawdust, water hyacinth and poultry litter biochar. Patwa et al.^62^ examinations of high plastic silt and clay sand amended with mesquite biochar showed similar results. Sun et al.^71^ analyses have also reported a reduction in MDD. These tests reveal that the bamboo biochar (BB) amendment adversely affects the MDD of BAS.

### Effect of biochar amendment on the UCS value of soil

**Figure 6:**
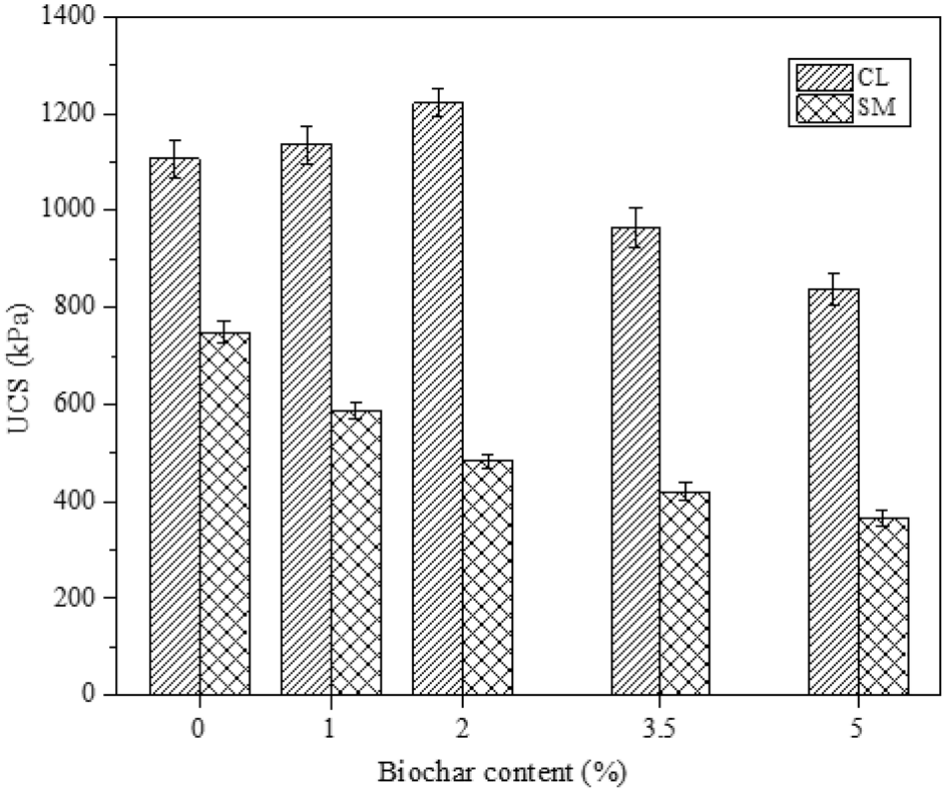
The effect of biochar increment (from 0 to 5% (w/w)) on UCS value of CL and SM soil.

**Figure 7:**
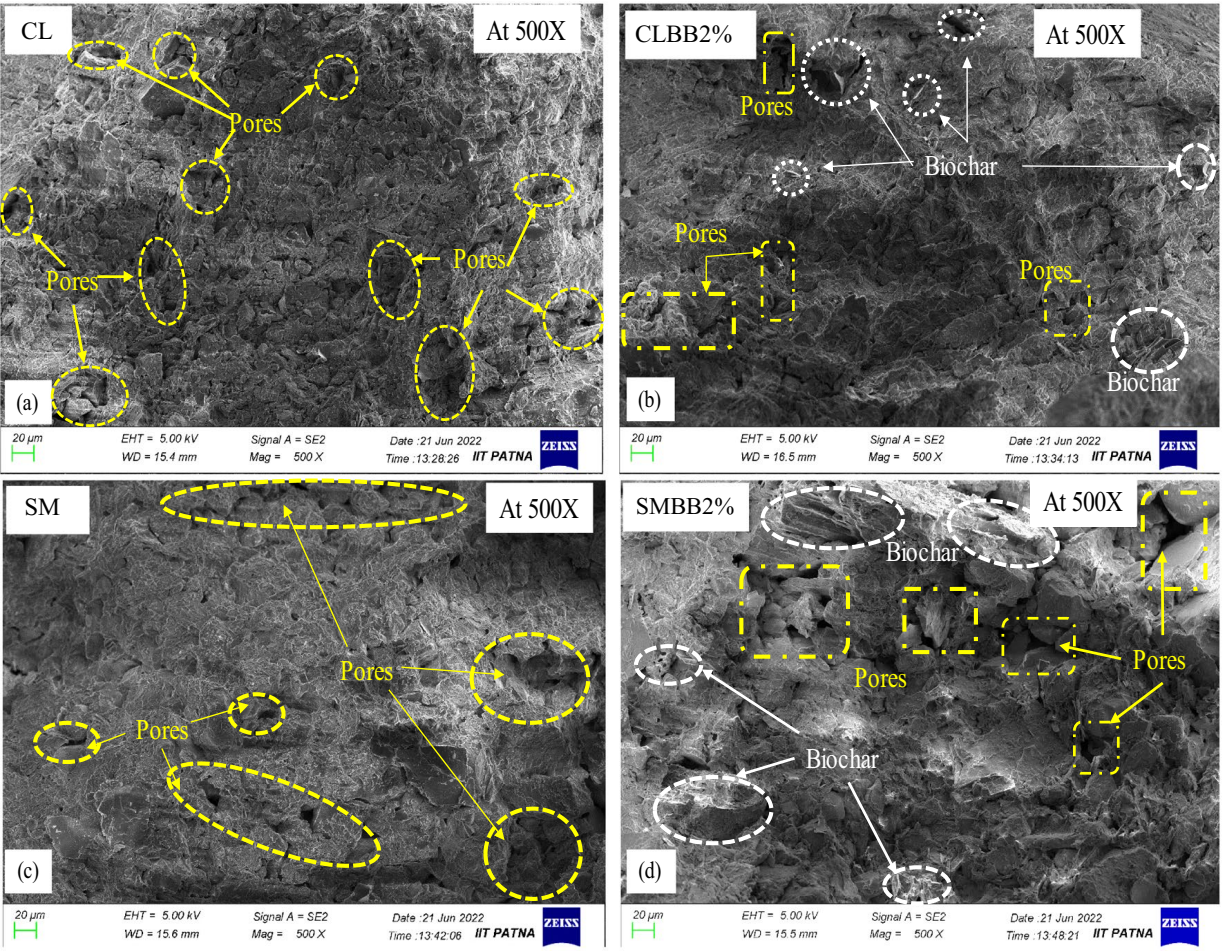
The change in pores and particle arrangement in the surface morphology of UCS specimens analysed at 500 magnification for (**a**, **c**) non amended CL, SM soil, and (**b**, **d**) CL, SM soil mixed with 2% (w/w) biochar content.

The unconfined compressive strength (UCS) result of CL and SM soils is presented in Fig. 6. The test performed by mixing 1% and 2% biochar content with CL soil resulted in an increase of 2.7% and 10.5% in UCS value compared to non-amendment soil (bare soil). The increase in UCS value can be attributed to the high surface roughness and frictional resistance of bamboo biochar. Furthermore, adding biochar content by 3.5% and 5% in the same soil causes a decrease in UCS value by 12.9% and 24.3%, respectively. Microscopic analysis performed on UCS specimens of; (a) non-amended and (b) amended soils is shown in Fig. 7. The analysis of FESEM images at × 500 magnification indicates that the 2% biochar (Fig. 7b) has filled more pores and better-clogged soil particles compared to the non-amended (Fig. 7a) specimen of CL soil. Thus microscopic analysis validates that the amendment of a lesser amount of biochar (2% BB) has filled more space, thereby providing better arrangement and interlocking, which leads to an increase in the UCS strength of CL soil. The increased UCS value suggests that the BB amendment strengthens the CL soil. It shows that BB and CL soil composite can be utilized as a landfill cover material. In contrast to the CL soil, the results of SM soil show a steady decrease in UCS value with an increment of biochar rate from 1 to 5%. Compared to non-amended SM soil, UCS values decreased by 21.5, 35.4, 43.8, and 51%, corresponding to 1, 2, 3.5 and 5% biochar content. The standard deviation of the UCS values obtained from biochar-amended CL and SM soils were found to be varying between 1.8 and 3.52% and 1.6–4.23%, respectively, as shown in Fig. 6. FESEM images of UCS specimens for non-amended and amended soils are shown in Fig. 7c,d. The microscopic images at × 500 magnification show that the 2% biochar (Fig. 7d) created more void spaces in the composite compared to the non-amended (Fig. 7c) specimen of SM soil. Moreover, biochar addition is attributed to larger void space and loss of soil-biochar integrity in SM soil. In addition, the functional group on the biochar surface enables the soil to hold more moisture, resulting in increased strain levels at peak stress, making soil composite more ductile. Higher water content lubricates the soil-soil and soil-biochar bonds and inhibits slippage between soil particles. Thus, BB inclusion resulted in the relaxation of soil-biochar integrity, thereby leading to an increase in the OMC, voids, and maximum strain at peak stress, accounting for reduced UCS value. Bora et al.^22^ studied on BAS with water hyacinth and sawdust biochar and showed reduced UCS value. However, the current study reveals that the UCS value was increased with CL soil (up to 2% BC) and decreased with SM soil on biochar (BB) amendment. The results showed that the UCS of BAS varies with types of soil. Moreover, Adhikari et al.^24^ have reported that the ageing of biochar gives physical stability to BAS. Therefore, the compressive strength of BAS would vary with the age of biochars.

### Effect of biochar on soil water retention characteristics

**Figure 8:**
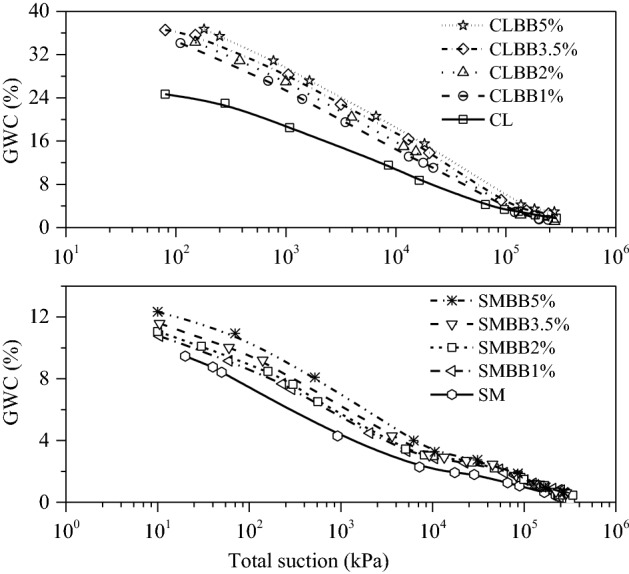
Gravimetric water content curves: for (**a**) CL, and (**b**) SM soil sample; each mixed with 0%, 1%, 2%, 3.5% and 5% (w/w) biochar content.

**Figure 9:**
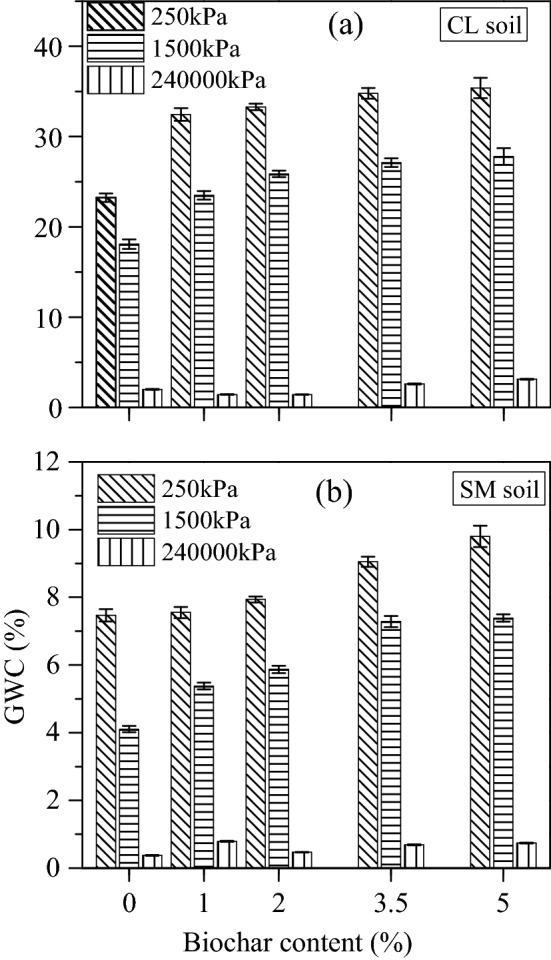
Variation of gravimetric water content (GWC) of (**a**) CL, and (**b**) SM soil; with the increment of biochar content (from 0 to 5% (w/w)) at 250 kPa, 1500 kPa and 240,000 kPa suction level.

Figure 8 shows soil water retention characteristics (SWRC) curve of BAS. The variation of gravimetric water content (GWC) with total suction of biochar amended CL (Fig. 8a) and SM soil (Fig. 8b) was observed. The test data presented in the suction range of 10–10^6^ kPa show that the GWC of both soils increased with the increment of biochar content. The increase was observed to be greater towards lower suction range (wet state) and lesser towards higher suction range (dry state). Furthermore, the GWC variations at different suctions (250 kPa, 1500 kPa and 240 × 10^3^ kPa) were also observed, as shown in Fig. 9. Generally, the GWC of soil at 1500 kPa signifies the permanent wilting point (PWP).

**Figure 10:**
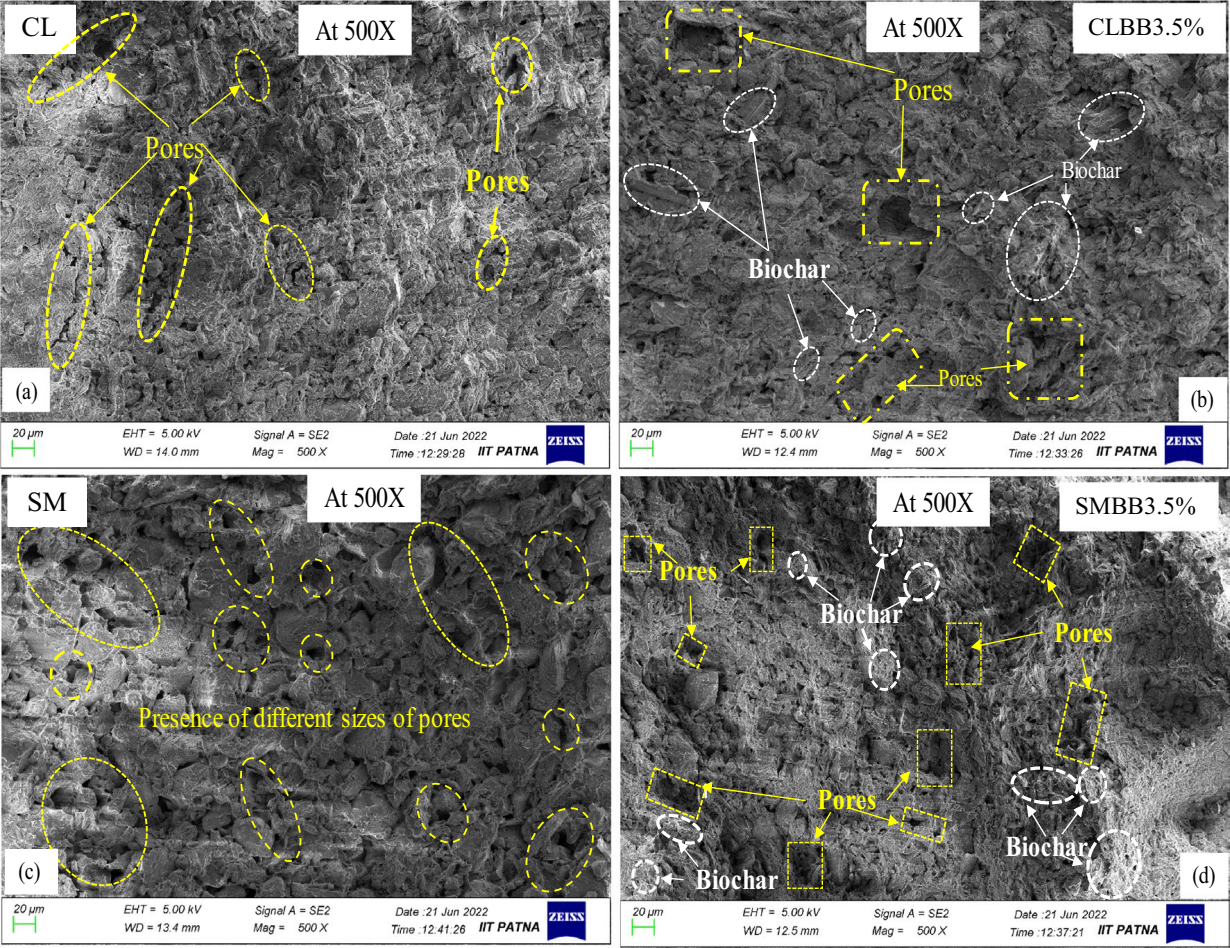
The change in number of pores and size in the surface morphology of WP4C specimens at 500 magnification: for (**a**, **c**) non amended CL, SM soil; and (**b**, **d**) CL, SM soil mixed with 3.5% (w/w) biochar content.

The analysis of SWRC curve of biochar amended CL soil (Fig. 9a) shows that, up on 1% biochar amendment, the GWC was noted to be increased by 9.2% at lower suction range (250 kPa) and decreased by 0.57% at higher suction range (240 × 10^3^ kPa) as compared to non-amended soil. Further mixing of 2% biochar, the GWC was noted to be increased by 9.9% at the lower suction range and decreased by 0.59% at the higher suction range. Similarly, at 3.5% and 5% biochar content, the GWC was noted to be increased by 11.56% and 12.15% at lower suction as well as 0.6% and 1.13% at higher suction, respectively. Moreover, the GWC was found to be increased by 4.7–9.72% at the PWP for 1–5% biochar content, respectively. The standard deviation of the mean GWC of biochar-amended CL soil was observed in the range of 0.23–2.95%. Due to legibility, the standard deviations are not included in Fig. 8. The increased WRC of BAS was attributed to the higher specific surface area (Table 1) and the hydrophilic nature of biochar. The high SSA of BB has increased the adsorbent surface in the composite, which enhanced the water retention property of BAS. Furthermore, microscopic images of non-amended (0% BC) and biochar amended (3.5% BC) WP4C specimens are shown in Fig. 10a,b for CL and in Fig. 10c,d for SM soil, respectively. The FESEM image depicted that the finer biochar particles reduced the diameter of the pores in the amended CL soil (Fig. 10b) more than the untreated case (Fig. 10a), which enhanced the capillarity phenomenon in the system. The increased capillary pores in BAS enhanced the water retention property of the sample. In addition to this, muscovite minerals (Fig. 3b) water absorption property also helped the water enhancement in the matrix.

Similarly, the results of SM soil (Fig. 8b) also showed that the WRC of BAS increased with biochar increment. In addition to that, a significant variation in GWC was noted over the suction range. The result shows that with higher biochar content (3.5–5%), an increase in GWC (1.43–2.33%) was observed at lower suction (Fig. 9b). Moreover, with 1–5% BC, the GWC was noted to be increased by 1.3–3.28% at PWP, respectively. The standard deviation of the mean GWC of biochar-amended SM soil was observed in the range of 0.12–3.5%. Due to legibility, the standard deviations are not included in Fig. 8. The standard deviations were observed comparatively more in lower suction than in higher suction ranges, as shown in Fig. 9. Furthermore, the FESEM images of biochar amended SM soil showed larger pores in the matrix (Fig. 10d) compared to the non amended case (Fig. 10c), which might be the reason for minor enhancement in WRC. The larger diameter pores affected the capillary in BAS, which resulted in less water retention capacity in the sample. Moreover, SM soil comprises predominantly quartz minerals with coarse grain size (Fig. 3c), which has minimal water holding capacity and affects the increase in water content in the matrix.

The interpretation of the SWRC curve shows that the water retention capacity of both soils increased with biochar addition. The WRC of BAS was noted to be improved with the increased quantity of BB in both soils. However, the increase in WRC of SM soil was lesser than that of CL soil with biochar increment. The microscopic image analysis showed that the difference in WRC was due to the alteration of pore sizes in both soils. The observation of FESEM images at × 500 magnification showed the reduction of larger pores and the increase of smaller pores in the biochar amended specimen compared to the untreated specimen for both soils. However, with a fixed amount of biochar (3.5%) amendment, the smaller diameter pores observed in CL soil were more than in SM soil. The smaller sizes of pores improve the capillary phenomenon in the BAS system. The rise of water in the capillary tube occurs through hydration and condensation. The modification in structural properties such as pore size and fineness in BAS has supported the water retention capacity of specimen^72^. Moreover, the CL soil was observed to have higher SSA than the SM soil and consisted of quartz and muscovite as predominant minerals. The CL soil has more clay content than SM soil, therefore, the high proportion of mesopores and micropores in BAS affected the WRC^24^. The higher SSA and water-absorbent minerals of CL soil have also supported the WRC in BAS. However, these observations marked that soil water retention capacity is not only affected by biochar content but also by soil types.

## Conclusions

The current study demonstrates the effect of bamboo biochar amendment (0, 1, 2, 3.5 and 5% by weight) on the compaction characteristics, mechanical properties (UCS) and water retention characteristics of CL and SM soil. Moreover, the index and physicochemical properties of untreated soil and BAS were also determined. Based on the findings of this investigation the following conclusions can be drawn:

The bamboo biochar addition decreased the specific gravity and increased liquid limits, plastic limits and pH of both soils. Biochar addition resulted in decreasing MDD and increasing OMC of both soils, however, the variation was observed to be higher for SM than CL type of soil. The change in these physical properties was mainly affected by the higher SSA, larger pore sizes and light weight of biochar. The addition of biochar was observed to increase the void space and air entrapment, leading to a decrease in the weight of the composite. Moreover, the water-holding nature of biochar created a slippery surface at the interface of soil-biochar composite leading to a decrease in MDD value.

The UCS value was found to increase in CL soil with the addition of BB, and the maximum value was observed at 2% biochar content. At the same time, the UCS value was found to be decreased with the further addition (3.5% and 5%) of BB in CL soil. The UCS value of BAS increased due to surface roughness, interlocking, frictional resistance, and filling of pore spaces with lesser biochar content. The increased UCS value indicates that the mixture of CL and BB can be used to strengthen the landfill cover. In contrast to biochar amended CL soil, a continuous decrease in UCS value was observed for biochar amended SM soil. Thus BB inclusion resulted in the relaxation of soil-biochar integrity, thereby leading to an increase in the OMC, voids, and maximum strain at peak stress, accounting for reduced UCS value.

The water retention capacity of both soils was found to be increased with the addition of biochar content. However, at the same biochar content, the WRC of CL was noted to be increased more compared to SM soil in slurry conditions. The reduction in larger pores and increase in smaller pores were observed more in biochar amended CL soil than in SM soil. It was observed that because of the capillarity phenomenon, the BAS sample with smaller pore sizes held more moisture than the sample with larger pores. Moreover, the increase in GWC on BAS was more towards lower suction range than that of the higher suction range. The increase in WRC was due to the clogging and entrapment of biochar particles in the soil matrix. The biochar amendment also increased GWC at the PWP in both soils, which would support vegetation growth. Hence, the landfill cover layer made with BAS will support vegetation growth. Therefore, it would also succeed in recovering landfill sites, particularly in bigger cities, where land scarcity is huge, and can be utilized for recreational and sports activities.

It is clear from the strength and water retention test that the properties of BAS soil vary with the types of soil and biochar content. Moreover, the increased UCS and WRC of BAS promise to be a suitable landfill cover material. However, further studies need to be conducted with different types of soil and BB by varying sample conditions for a better understanding of the behaviour of biochar amendment soil. Further investigations on aged biochar are required to understand biochar amendment soil’s mechanical and hydrological properties.

## Supplementary Information

Supplementary Information 1.

Supplementary Information 2.

## Data Availability

All data generated or analysed during this study are included in this published article [and its supplementary information files].
